# Special Issue “Zebrafish-A Model System for Developmental Biology Study”

**DOI:** 10.3390/jdb8030015

**Published:** 2020-08-04

**Authors:** Lisa Maves

**Affiliations:** 1 Center for Developmental Biology and Regenerative Medicine, Seattle Children’s Research Institute, 1900 Ninth Avenue, Seattle, WA 98101, USA; lmaves@u.washington.edu; 2 Division of Cardiology, Department of Pediatrics, University of Washington, Seattle, WA 98105, USA

**Keywords:** zebrafish, calcium imaging, muscle, ethanol, pigment cell, protein kinase A signaling, dietary regimen, eye, morphogenesis, autism spectrum disorders

For this Special Issue “Zebrafish-A Model System for Developmental Biology Study,” we present a collection of studies, including original research papers and review articles, that focus on advances in developmental biology research and that take advantage of the zebrafish model organism. Zebrafish are a prominent system for obtaining a basic research understanding of developmental biological processes, and the use of zebrafish as an animal model for human disease continues to grow. The articles in this Special Issue underscore the diversity and utility of zebrafish research for basic and biomedical developmental biology.

This Special Issue contains three original research articles that report on a variety of areas of developmental biology. Bergmann et al. [1] developed approaches to perform live imaging of neuronal activity in the optic tectum of the brain in late-stage zebrafish larvae. They developed a new transgenic zebrafish strain (*NBT:GCaMP3*) that provides robust expression of a fluorescent calcium reporter in all major areas of the brain in up to 21 day-old larvae. The authors were able to use this transgenic strain to characterize receptive fields of neurons in the optic tectum at later larval stages than had been previously possible. This new transgenic strain will provide opportunities to further examine the connections between neural activity and behavior in developing zebrafish larvae. Coffey et al. [2] examined the effects of ethanol exposure on skeletal muscle development in zebrafish larvae. As in the Bergmann et al. study, Coffey et al. focused on the understudied stages of larval development. Coffey et al. found that ethanol treatments in zebrafish larvae caused muscle degeneration, likely by inducing cellular stress and muscle cell membrane damage and by disrupting muscle fiber adhesion to the extracellular matrix. A previous study showed that ethanol exposure during earlier zebrafish embryonic muscle development led to smaller muscle fibers but not muscle degeneration [3]. Thus, these studies showed that zebrafish can provide a model for understanding the pleiotropic effects of ethanol exposure at different developmental stages. Cooper et al. [4] showed that the protein kinase A signaling pathway inhibits the differentiation of a specific type of pigment cell, iridophores, in zebrafish larvae. They also found that protein kinase A signaling may simultaneously promote the development of a subset of a different pigment cell type, melanophores. This work helps to illuminate the pathways that regulate the specification and differentiation of subsets of pigment cells that develop from neural crest cells. These three research articles all take advantage of multiple experimental approaches, each employing genetics, transgenic strains, chemical treatments, and advanced imaging techniques. Together, these papers exemplify the value of applying diverse accessible approaches in zebrafish developmental biology research.

A fourth original research article from Monteiro et al. [5] examined the effects of different feeding regimens on zebrafish growth, survival, and reproductive performance. Even though zebrafish is a well-established animal model, there is still much variation across zebrafish facilities in husbandry methods, such as feeding protocols. How feeding regimens impact zebrafish development, or the penetrance and expressivity of mutant and disease phenotypes, is an understudied and often underappreciated aspect of zebrafish research [6]. An interesting finding from Monteiro et al. came from their comparison of two different commercially available processed feeds. They found that one feed improved survival rate and embryo viability, whereas a second feed enhanced growth. The work from Monteiro et al. thus provided insight into and, hopefully, stimulates a greater appreciation for the impacts of feeding protocols on zebrafish biology.

This Special Issue also contains two review articles. Cavodeassi [7] provided a review of vertebrate eye morphogenesis, focusing on the roles of cell shape and tissue rearrangements that have been illuminated in zebrafish and in another fish model, medaka. Kozol [8] reviewed how zebrafish provides an animal model for understanding the neurodevelopmental disorders autism spectrum disorder and intellectual disability. In particular, this review focuses on how zebrafish can provide insight into the developmental basis and prenatal factors that contribute to these disorders. Both of these reviews are very comprehensive and feature extensive reference lists. These reviews should stimulate future zebrafish studies in both of these research areas.

I would like to thank all of the contributing authors for presenting their work in this Special Issue. I would also like to thank the reviewers for their evaluations of the submitted articles, the Special Issue co-editors Mark W. Majesky and Przemko Tylzanowski, and the editorial staff at the Journal of Developmental Biology for their efforts in assembling this Special Issue.
